# Pathology of Inflammatory Diseases, &c. Founded on the Blood's Vitality

**Published:** 1825-07

**Authors:** John Jones


[To be continued.]

---

ART. II.—*Pathology of Inflammatory Diseases, &c. founded on the Blood's Vitality.* By JOHN JONES, Esq.

[Continued from vol. liii. page 592.]

THE second class of diseases proposed to be considered in connexion with the blood's vitality, and as illustrative of the preceding pathological views, are those of increased arterial action. I have endeavoured to prove that venous congestion is necessarily productive of debility, and greatly tends to modify diseases; many of which are accompanied with diminished power, in a ratio in some measure proportionate to the extent of the congestions, the particular organs so affected, and the suddenness of their production. Arterial fulness, on the contrary, is attended with increased vigour, which constitutes a prominent character of the diseases with which it is connected. These may be considered under the following heads:—1. Inflammatory diseases, or phlegmasiæ of Cullen; 2. Hectic fever.

1. *Inflammatory Diseases.*—The phenomena of inflammation were explained in a former paper, as depending exclusively on arterial action. It was then mentioned, that as the principle of vitality, or *materia vitæ diffusa*, is chiefly formed in arterial blood, the energies of the part affected with inflammation become preternaturally augmented: the nerves are rendered morbidly sensible, and an expenditure of nervous energy occurs, in proportion to the increased vascular action. It was also observed, that, when the inflammation is great, or affects an important organ, the system partakes of the morbid excitement, and sympathetic fever is produced.

The most frequent cause of inflammation of the internal organs, is checked perspiration from exposure to cold. The influence of cold, conjoined with a damp atmosphere, is the prolific source of many of the most fatal diseases prevalent in our variable climate. The secretion of perspiration, like all other organic functions, is supported by a free circulation of arterial blood in the secreting organ. If, from any cause in the cuticular organisation, this is prevented, perspiration is deficient, and the temperature of the skin diminished. In inflammatory action, and those fevers in which arterial excitement is well marked by great heat and dryness of the skin, there is reason to believe, with Dr. Darwin, that perspiration is no longer obstructed, but increased, and, owing to the rapid production of caloric, and augmented activity of the absorbents, is carried off as soon as it becomes formed. The perspiration not only preserves the skin in a soft and pliable state, but removes from the circulation what, if retained, would prove noxious. The functions of the skin, therefore, are of the utmost importance in the animal economy, and any interception to their due performance necessarily leads to disease. Cold, particularly when conjoined with moisture, is undoubtedly a sedative agent, and its primary and direct effect is to depress the nervous power. By its influence, the secreting vessels of the skin are deprived of their wonted energies, and perspiration becomes obstructed; the blood is, in a measure, driven from the surface, and accumulates in the internal organs, producing congestion, which is an additional cause of nervous depression: a consequent morbid accumulation of vitality occurs in the arterial blood, and becomes expended, by producing inflammatory action in one or other of the viscera, according to the existing predisposition for such morbid action. The proximate cause of inflammation, therefore, is similar to that of idiopathic fever, and consists in diminished nervous power. The difference between them is, that in one the local and constitutional excitement, by preternaturally augmenting the energies of the system, overcome the depressing agent. In the other, the debilitating power continues its influence through the whole course of the disease, and the vascular action, although morbidly increased, is weak, and attended with corresponding diminished tone in the system. As inflammation and idiopathic fever are alike produced by nervous depression, there is reason to believe that the occurrence of one is frequently prevented by the existence of the other; or that topical inflammation prevents idiopathic fever, which would otherwise be produced, *et vice versâ.*

It not unfrequently happens, in inflammatory diseases, that the accompanying febrile excitement continues long after the local affection is removed, and, if not restrained by judicious treatment, might exhaust the vital energies. The continuance of febrile action, from whatever cause produced, appears to be the effect of loss of balance between the venous and arterial circulations; and therefore, till this equilibrium is restored, the fever does not wholly subside, and, to a casual observer, the disease appears unabated.

*Treatment.*—The mode of treatment usually pursued in the phlegmasiæ, is agreeable to the above principles, and consists in lowering the system till the inflammatory action is subdued. For this purpose, the practitioner's chief dependence is upon blood-letting; and the rule for abstracting blood is, not to be guided by the quantity, but the effects it produces on the system. As arterial blood is principally concerned in the production of inflammation, the abstraction of a much smaller quantity of it would appear to have a more beneficial effect than a larger quantity of venous blood; and, therefore, the practice of opening a vein in such cases seems to have nothing to recommend it, but convenience to the operator. That this is the fact, is proved by the very decided benefit derived from arteriotomy in phrenitis; and likewise the application of leeches or cupping-glasses in most cases of internal inflammation, in each of which operations comparatively little blood is removed, and yet with the best effects.

When febrile excitement continues after the local affection has subsided, opiates are found to be highly beneficial. They restrain the vascular disturbance, tranquillise the system, and afford an opportunity of supporting the weakened energies by more nutritive diet, &c. than would be admissible in the more early stage of the disease.

*Hectic Fever*—is the effect of continued, but not violent, local action, produced by an irritating cause, which the constitution is unable to overcome. It generally accompanies those disorganisations produced by what is called scrofulous inflammation, particularly when affecting internal organs or the larger joints. It is also an attendant on large suppurating wounds, which are prevented from speedily healing, as in compound fractures, &c. and soon exhausts the powers of life, unless the irritating cause be removed. Like sympathetic fever accompanying the phlegmasiæ, therefore, hectic depends upon local irritation, in which the energies, not only of the part affected, but also of the whole system, become preternaturally augmented and expended. The symptoms, however, are very different. The local irritation in hectic is, for the most part, ulceration slowly formed, tardy in its progress, and, although not violent, continued in its operation. The effects on the constitution are consequently insidious, gradual, and permanent, and the fever is remarkably remittent, with regular and distinct exacerbations. The inflammation producing sympathetic fever is phlegmonous, suddenly induced, intense in its action, and rapid in its progress; and the consequent fever is continued, corresponding in violence to the extent of the local affection on which it depends. The tendency of fevers to remit, is attributed by Cullen to the diurnal revolutions which occur in the operations of the body in a state of health. Hectic fever seems peculiarly subservient to this law in the animal economy: sympathetic fever, on the contrary, is subject to its influence only in a slight degree, in consequence of the violent action by which it is characterised.

A predisposition to hectic is frequently constitutional, and in some measure hereditary: thus, we find phthisis pulmonalis affecting many individuals of the same family. Such predisposition seems also particularly connected with a sanguineous temperament, which may be distinguished by the following characters:—a smooth, fair skin, with or without a florid complexion, light hair and eyes, a remarkable disposition to inflammatory complaints, which have a tendency to be rapid in their progress. The mind, partaking also of its influence, is peculiarly susceptible of impressions of every description; the imagination is acute and lively; the disposition marked by ingenuousness, vivacity, and good humour; the passions are readily excited, and, unless restrained by reason and moral principle, are perpetually disposed to run into excess. These peculiarities are doubtless intimately connected with arterial plethora, which not only produces its peculiar effects on the system when in health, but tends greatly to modify disease. In the sanguineous temperament, the primary symptoms of hectic are easily excited, even without being preceded by any evident local irritation: it is, however, complete only when the irritating cause is great, and of a permanent description. The phthisical patient is subject to hectic flushes, attended with an accelerated pulse, from the most trivial excitement, long before symptoms of diseased lungs occur. At length, probably from exposure to cold, pulmonary irritation is produced, tubercles become formed; or, perhaps, having previously existed in a dormant state, are excited into action. By appropriate remedies, the local irritation subsides, till again excited by fresh exposure to cold; although such patients are seldom altogether without cough, which is generally dry, and accompanied with a quickened pulse. At every repetition of the exciting cause, there are daily slight exacerbations of fever. These symptoms, which are the beginning of hectic, might be allayed, and the occurrence of ulceration, on which confirmed phthisis depends, be protracted from time to time, by judicious treatment. But, sooner or later, the evil hour arrives, when the permanently accelerated pulse, daily exacerbations of fever, purulent expectoration, general emaciation, and colliquative sweats and diarrhœa, loudly declare to all but the patient that a termination to his terrestrial career is rapidly approaching. The general state of excitement, so strongly marked in phthisis, is a remarkable illustration of the connexion between the mind and circulation. The patient, for the most part, possesses comparative tranquillity and cheerfulness, and is buoyed up with hope nearly to the last moment of his existence, even against his own better judgment, and the convictions of his surrounding friends and medical attendants.

*Treatment.*—As hectic fever depends wholly on a local affection, which is generally beyond the reach of medicine, our prognosis must uniformly be unfavourable. Ulceration in any of the internal organs rarely admits of cure, and the constitutional derangement which accompanies it terminates only with the life of the patient. That this is the case in phthisis pulmonalis, we have daily and abundant proof. Hopeless, however, as such cases are, solitary instances occasionally occur in which recoveries have been complete, even when the lungs have been in a state of ulcerative disorganisation. According to Laennec, whose opportunities for tracing the effects of disease by post-mortem examinations appear to have been very numerous, this is by no means so uncommon as is generally believed. We should, therefore, not despair, even when the prospect is most discouraging. The indications are—1, to remove, if possible, the local irritation; 2, to allay the consequent constitutional disturbance, and to support the impaired energies of the system by the least stimulating means; 3, to check urgent symptoms, as colliquative sweats and diarrhœa, immediately as they arise.

I shall only observe on the means which are usually employed for fulfilling the above indications, that counter-irritation appears most entitled to confidence for overcoming the local disease on which hectic fever depends. For this purpose, the constant repetition of blisters, a perpetual blister, or seton, applied as nearly to the part affected as possible, should never be omitted. When disorganisation has only commenced, and not extended its influence too far, it might be arrested, and the diseased organ restored to its functions, by such measures duly persevered in. The following case is illustrative of the beneficial effects derived from counter-irritation.

Mr. T. æt. about fifty, of a spare habit and slender make, was attacked with febrile symptoms, which made their approach insidiously, and assumed the character of the prevailing epidemic. By the treatment pursued, in a few weeks he appeared convalescent, and debility only remained. This state of debility, however, continued to increase for some time, without an apparent cause, and, in about a month from the cessation of the fever, a new train of symptoms supervened. His cough, which had been rather troublesome from the commencement, was now increased, with a copious expectoration, which appeared purulent. Pulse permanently accelerated, attended with evening exacerbations of fever, profuse colliquative sweats, and great emaciation. By the suggestion of the physician in attendance with myself, a seton was introduced on the chest. Soon after it began to suppurate, amendment was evident: his cough became less troublesome, the expectoration improved in its appearance, the nocturnal sweats ceased, and the febrile excitement gradually subsided. In the course of a few months, his health was perfectly restored, and he now feels better than he has done for some years. The medicines employed were chiefly intended to allay irritation, and consisted of demulcents, conjoined with antimonials and hyosciamus; together with the occasional use of gentle aperients.

[To be continued.]

